# Compatibility of whole-genome sequencing data from Illumina and Ion Torrent technologies in genome comparison analysis of Listeria monocytogenes

**DOI:** 10.1099/mgen.0.001389

**Published:** 2025-05-01

**Authors:** Stefanie Lüth, Jannika Fuchs, Carlus Deneke

**Affiliations:** 1 Department of Biological Safety, German Federal Institute for Risk Assessment, Max-Dohrn-Str. 8-10, 10589 Berlin, Germany; 2 Chemical and Veterinary Investigation Office (CVUA) Karlsruhe, Weißenburger Str. 3, 76187 Karlsruhe, Germany

**Keywords:** core genome multilocus sequence typing (cgMLST), genomic surveillance, outbreak investigation, single nucleotide polymorphism (SNP)

## Abstract

Whole-genome sequencing (WGS) has become the key approach for molecular surveillance of *Listeria monocytogenes*. Genome comparison analysis can reveal transmission routes that cannot be found with classic epidemiology. A widespread standard for use in genome comparison analysis involves data from short-read sequencing, generated on Illumina or Ion Torrent devices. To date, little is known about the compatibility of data from both platforms. This knowledge is essential when it comes to the central analysis of data, for example, in the case of outbreaks. We used WGS data from 47 *L*. *monocytogenes* isolates of the strain collection of the German National Reference Laboratory for *L. monocytogenes*, generated on either Illumina or Ion Torrent devices, to analyse the impact of the sequencing technology on downstream analyses. In our study, only the assembler SPAdes delivered qualitatively comparable results. In the gene-based core genome multilocus sequence typing (cgMLST), the same-strain allele discrepancy between the platforms was 14.5 alleles on average, which is well above the threshold of 7 alleles routinely used for cluster detection in *L. monocytogenes*. An application of a strict frameshift filter in cgMLST analysis could push the mean discrepancy below this threshold but reduced discriminatory power. The impact of the platform on the read-based single nucleotide polymorphism analysis was lower than that on the cgMLST. Overall, it was possible to improve compatibility in various ways, but perfect compatibility could not be achieved.

**Impact Statement**

Genomic surveillance plays an important role in protecting consumers worldwide from foodborne pathogens. In this context, often decentrally generated whole-genome sequencing (WGS) data need to be combined in a comprehensive analysis to provide a national or international picture of possible transmission pathways. One challenge is that WGS data can be generated in many different ways, for example, using different sequencing technologies. To perform reliable analyses, we need to know how compatible the data are with each other. Our study provides an assessment of what needs to be considered when analysing data from Illumina and Ion Torrent sequencers together. Using real-world outbreak examples, we illustrate the difficulties, as well as the opportunities, of combining data from both sequencing platforms to provide a data-driven assessment for surveillance users.

## Data Summary

The authors confirm all supporting data, code and protocols have been provided within the article or through supplementary data files.

## Introduction

In recent years, whole-genome sequencing (WGS) has become the key approach for molecular surveillance of foodborne bacterial pathogens [1,2]. Decreasing costs and increasing efficiency (higher throughput and shorter runtimes) have opened the doors for its widespread application [3,4].

In the case of listeriosis, which is caused by the foodborne pathogen *Listeria monocytogenes*, it is difficult to recognize outbreaks as such and to identify their source. Typically, listeriosis cases occur over a wide area in time and space. The severity of the disease, the variable incubation period and the wide range of foods involved make it difficult to draw conclusions about possible outbreak vehicles from patient surveys. Genetic similarities are used to generate hypotheses about transmission routes that would not be possible using classical epidemiology. WGS has become indispensable in the investigation of listeriosis outbreaks in Germany [5,8] and worldwide [9,10]. In the long term, the use of WGS can reduce listeriosis outbreaks, which is a major public health benefit [11].

The widely used standard for WGS to perform genome comparisons is short-read sequencing. Its main advantage over long-read sequencing is the higher confidence in base calls due to the high coverage [3]. In a 2016 survey by the European Centre for Disease Prevention and Control, the vast majority of respondents from EU/EEA countries reported access to Next Generation Sequencing technologies [12]. In the EU and worldwide, Illumina instruments are the most commonly used for short-read sequencing [12,14]. Ion Torrent instruments are less commonly used, but they also play an important role in short-read sequencing.

Both technologies use the principle of sequencing by synthesis, but they differ in how incorporated bases are detected. Illumina sequencing detects fluorescence signals optically [15], whereas Ion Torrent sequencing detects pH changes using semiconductor technology [16]. Other differences include read lengths and runtimes. Each technology has its advantages and disadvantages, and it is up to the users to decide what is most suitable for their purposes. In Ion Torrent sequencing, runtimes are usually shorter, and the resulting reads are longer than those in Illumina sequencing [17]. However, only single-end mode is available, and homopolymer errors (e.g. insertions and deletions) are frequent [18,19]. In two independent sequencings of the same isolate, the same homopolymer errors do not occur [20,21], making it difficult to find and filter them out.

It is a significant step forward that local analyses of bacterial isolates are possible due to the affordability of WGS. Systems such as the EFSA One Health WGS System [22] or the NCBI Pathogen Detection Pipeline [23] have been established to keep track of the spread of bacterial pathogens. However, it also represents a challenge to carry out comprehensive and precise cluster analyses from the locally obtained data [10].

Depending on the study and the type of downstream analysis, the results were found to be more or less comparable between Illumina and Ion Torrent platforms. Negligible differences were found when detecting antimicrobial resistance genes or performing single nucleotide polymorphism (SNP) analysis for bacterial sequences [21,24], whereas larger differences were found in core genome multilocus sequence typing (cgMLST) analysis [21]. In Germany, there were two proficiency tests in which sequence data generated in different laboratories were compared with each other [21,25]. In the first-round proficiency test [21], the laboratories were given DNA for sequencing, and the second one [25] started with bacterial cultures. Some laboratories used Ion Torrent devices for sequencing, which provided the first insights into the differences in sequence data from Illumina technology. Overall, the SNP results were more comparable than the results from the cgMLST analysis, and frameshifts in the Ion Torrent data were identified as the main cause. However, the datasets were small, and no in-depth root cause analysis was performed.

To date, there are still wide knowledge gaps on the compatibility of WGS data from Illumina and Ion Torrent devices. Therefore, our study aimed to compare data from both technologies, especially with regard to the impact on downstream analyses for genome comparison, such as cgMLST and SNP analysis. Our study reveals differences, traces their origin and provides application-oriented guidance for evaluating analyses from mixed sequence data.

## Methods

### Strain set

A total of 47 *L*. *monocytogenes* isolates sampled between 2015 and 2020 in official controls in Germany were included in the study. They originated from different foods (mainly meat and fish products), food-processing environments or animal feed. Nine of the isolates have been published as part of German listeriosis outbreaks [5,7].

### Illumina sequencing

*L. monocytogenes* isolates were incubated on Sheep Blood Agar overnight at 37 °C. The PulseNet protocol for Gram-positive bacteria was used for cell lysis (https://www.cdc.gov/pulsenet/pdf/pnl32-miseq-nextera-xt.pdf), followed by DNA extraction using the QIAmp DNA Mini Kit (Qiagen, Hilden, Germany) or the PureLink Genomic DNA Mini Kit (Invitrogen™, Carlsbad, CA, USA). DNA was quantified using the Qubit dsDNA BR Assay Kit with the Qubit™ 2.0 Fluorometer (Invitrogen, Carlsbad, CA, USA). The sequencing library was prepared with the Illumina Nextera XT DNA Library Kit or the DNA Prep Kit (Illumina Inc., San Diego, CA, USA). Sequencing was performed in the paired-end mode with 2×150 bp using the Illumina NextSeq 500 or with 2×300 bp using the Illumina MiSeq (Illumina Inc., San Diego, CA, USA).

### Ion Torrent sequencing

*L. monocytogenes* isolates were incubated in Brain Heart Infusion Bouillon overnight at 37 °C. DNA was extracted using the Maxwell Cultured Cells Kit (Promega, Madison, WI, USA), quantified using the Qubit dsDNA BR Assay Kit with the Qubit 2.0 Fluorometer (Invitrogen) and fragmented mechanically. The sequencing library was prepared with the Ion Plus Fragment Library Kit (Thermo Fisher Scientific, Waltham, MA, USA). Sequencing was performed using the Ion 510, Ion 520 and Ion 530 Kit-Chef (Thermo Fisher Scientific) on the Ion Torrent S5 instrument (Thermo Fisher Scientific). The Ion Torrent suite version 5.12.1 was used.

### Trimming, assembly and quality control

The raw sequences were trimmed, assembled and quality-checked with the pipeline AQUAMIS [26]. The pipeline was used with three different assemblers: MEGAHIT [27], SKESA [28] and SPAdes [29].

### Core genome multilocus sequence typing

cgMLST was performed with the pipeline chewieSnake [30]. A cgMLST scheme containing 1,748 loci [31] was used.

#### Frameshift filtering

As Ion Torrent data are known to be prone to homopolymer errors, which can lead to frameshifts [18,19], we investigated the influence of filtering allele calling results with two different frameshift filters. Frameshift filters test the length of each allele and compare them with the median length of all alleles of a locus. The relative frameshift filter (f.s.r.) removed alleles if they deviated by a relative fraction (0.1) to the median length. The absolute frameshift filter (f.s.a.) removed alleles if they deviated by an absolute difference (9 bp) to the median length. We focused on SPAdes assemblies only as these were shown to produce the only reliable assemblies for Ion Torrent data.

#### Sensitivity and specificity of cluster attribution

To create hypotheses on possible epidemiological relatedness, clustering of *L. monocytogenes* isolates was performed using a threshold value of ≤7 alleles for the pairwise distance [31]. Single-linkage clustering was applied to a cgMLST distance matrix to determine the number of sample pairs clustering within this threshold with either platform. Clustering results from Illumina data assembled with SPAdes were defined as the clustering truth. On that basis, the correctness of clustering based on Ion Torrent data was assessed as follows:

- True positives (TP): both ≤7 allele differences (AD);
- True negatives (TN): both >7 AD;
- False positives (FP; false claim by Ion Torrent): Illumina>7 AD, Ion Torrent≤7 AD;
- False negatives (FN; missed by Ion Torrent): Illumina≤7 AD, Ion Torrent>7 AD.

Sensitivity [TP/(TP/FN)] and specificity [TN/(FP/TN)] after the use of different frameshift filters (none, f.s.r. and f.s.a.) were calculated.

#### Test with known outbreak-related isolates

Nine isolates were known to be linked to six listeriosis outbreaks, including epidemiological confirmation [5,7]. The publicly available sequence of one clinical isolate per outbreak was selected as a reference to search for matches (distance ≤7 alleles in cgMLST) in the study dataset. Those clinical reference sequences were sequenced on Illumina platforms. For accessions of references, see Table 1.

**Table 1.** Non-clinical isolates known to be linked to listeriosis outbreaks and their allele distance to the reference sequence from a clinical isolate. The accession of the reference sequence is given below the cluster name. Either no filter (none), an f.s.r. or an f.s.a. was used in cgMLST analysis. A distance threshold of ≤7 alleles is commonly used to identify matches between clinical and non-clinical isolates. Distances above this threshold are marked in red

Listeriosis cluster	Sample ID	Platform	None	F.s.r.	F.s.a.
Beta1 (ERS4418848)	16-LI00360-0	Illumina	1	1	1
		Ion Torrent	8	5	2
	18-LI00760-0	Illumina	2	2	2
		Ion Torrent	14	10	3
Delta1 (ERS2103330)	16-LI00426-0	Illumina	1	1	1
		Ion Torrent	13	6	0
Epsilon1a (ERS3572660)	19-LI00138-0	Illumina	0	0	0
		Ion Torrent	19	12	2
	19-LI00175-0	Illumina	0	0	0
		Ion Torrent	12	6	1
Rho3 (ERS4366826)	16-LI00918-0	Illumina	4	4	4
		Ion Torrent	22	16	5
	16-LI01165-0	Illumina	2	2	2
		Ion Torrent	3	2	2
Zeta5a (ERS7696627)	18-LI00595-0	Illumina	1	1	1
		Ion Torrent	28	15	5
	18-LI00596-0	Illumina	0	0	0
		Ion Torrent	34	20	2

#### Cause analysis for discrepancies between Illumina and Ion Torrent

In order to identify reasons for discrepancies between cgMLST results from Illumina and Ion Torrent, all allele sequences of loci, where allele numbers differed between Illumina and Ion Torrent, were collected, again focusing on SPAdes assemblies only. We performed a global pairwise sequence alignment on these allele sequence pairs to identify insertions, deletions, mutations and other missing bases using the Biostrings R package [32].

### SNP analysis

SNP analysis was performed with the pipeline snippySnake [33]. The annotated genome of the *L. monocytogenes* reference strain EGDe (NC_003210.1) was used as a reference. Trimmed read data from the AQUAMIS pipeline [26] was used as input. We analysed three different datasets: Illumina data only, Ion Torrent data only and Illumina and Ion Torrent data combined.

## Results

### Study population structure

The 47 *L*. *monocytogenes* were from serogroups IIa (*n*=21), IIc (*n*=12), IVb (*n*=7), IIb (*n*=6) and IVa (*n*=1). The isolates had 18 different MLST CCs, and the 10 most frequent ones were CC9 (*n*=12), CC6 (*n*=5), CC5 (*n*=4), CC37 (*n*=3), CC121 (*n*=3), CC8 (*n*=3), CC11 (*n*=3), CC20 (*n*=2), CC29 (*n*=2) and CC26 (*n*=2).

### Quality control comparing different assemblers

**Fig. 1.**
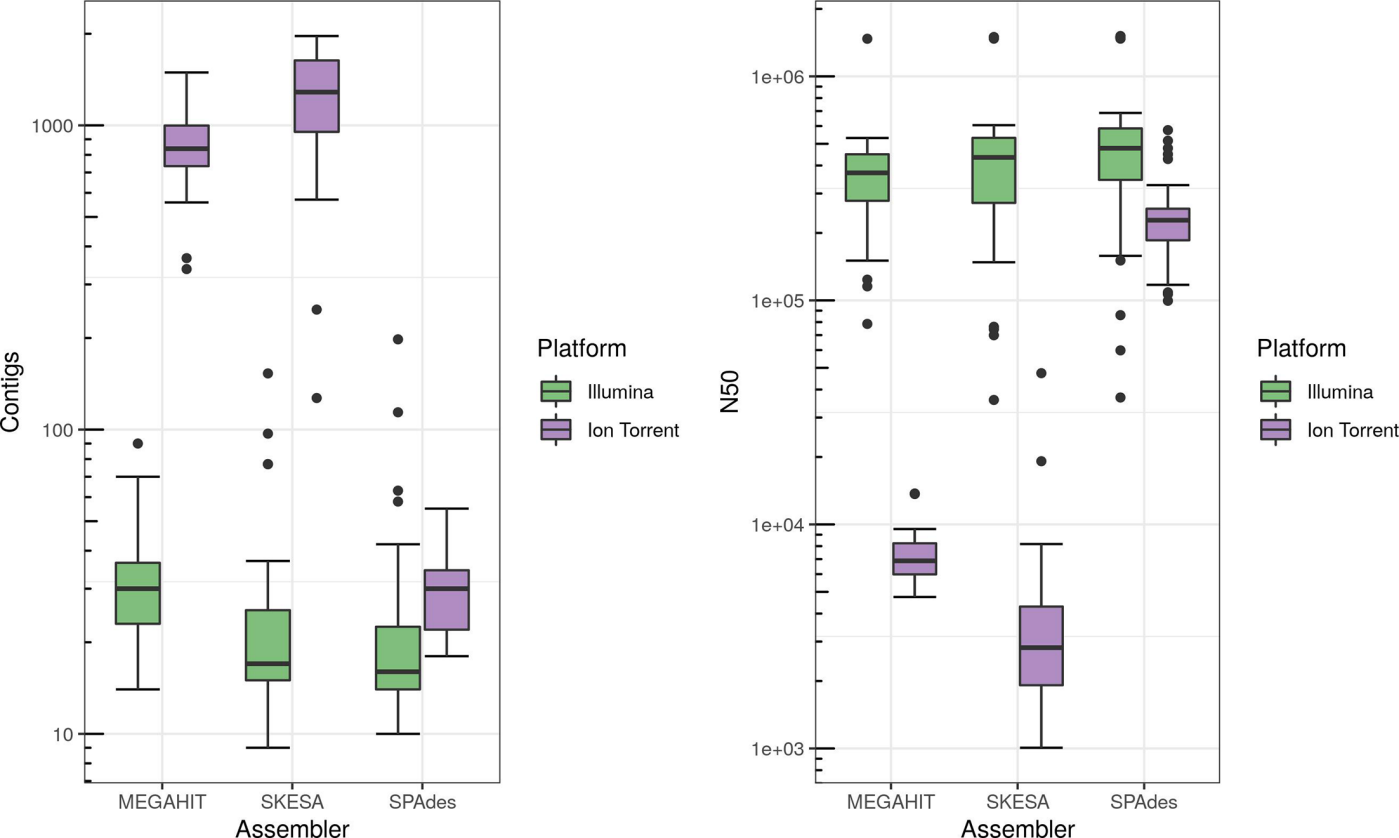
Assembly quality of sequencing data from Illumina and Ion Torrent using different assemblers. Left: number of contigs; right: N50.

CgMLST is based on the pairwise comparison of genes. The assemblies from the three different assemblers were checked for the number of contigs and the N50 as parameters of completeness (Fig. 1). Our assumption here was that the more complete the assembly, the more likely it is that the cgMLST loci in the assembly have been successfully assembled and could be analysed.

The number of contigs was lower, and the N50 value was higher for Illumina than for Ion Torrent data, independent of the assembler, which represents the smallest difference between the platforms that appeared for SPAdes assemblies.

**Fig. 2.**
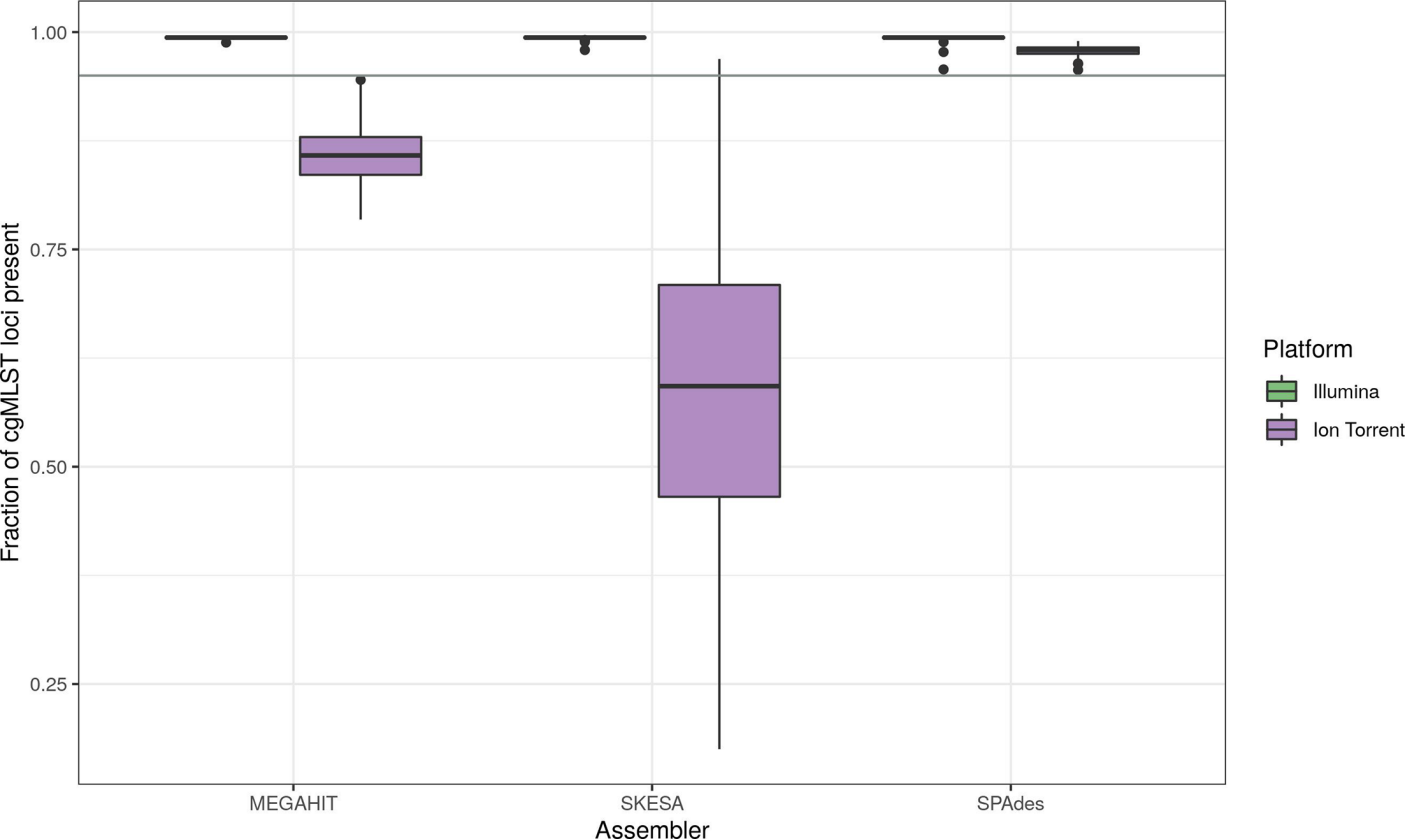
Fraction of cgMLST loci found after the assembly of Illumina and Ion Torrent data with different assemblers. The horizontal grey line indicates a threshold value of 0.95.

The fraction of cgMLST loci found (no frameshift filter applied) was higher for Illumina than for Ion Torrent data (Fig. 2).

The mean number of missing loci for Illumina data was independent of the assembler (between 11 and 13 loci) but ranged from 38 (SPAdes) over 255 (MEGAHIT) to 730 (SKESA) for Ion Torrent data. The smallest difference between the platforms appeared for SPAdes assemblies. Only cgMLST results from SPAdes assemblies met the minimum quality requirement of at least 95% of loci present and were thus used for the following analyses.

### cgMLST analysis based on SPAdes assemblies, with and without frameshift filtering

#### Same-strain discrepancy

**Fig. 3.**
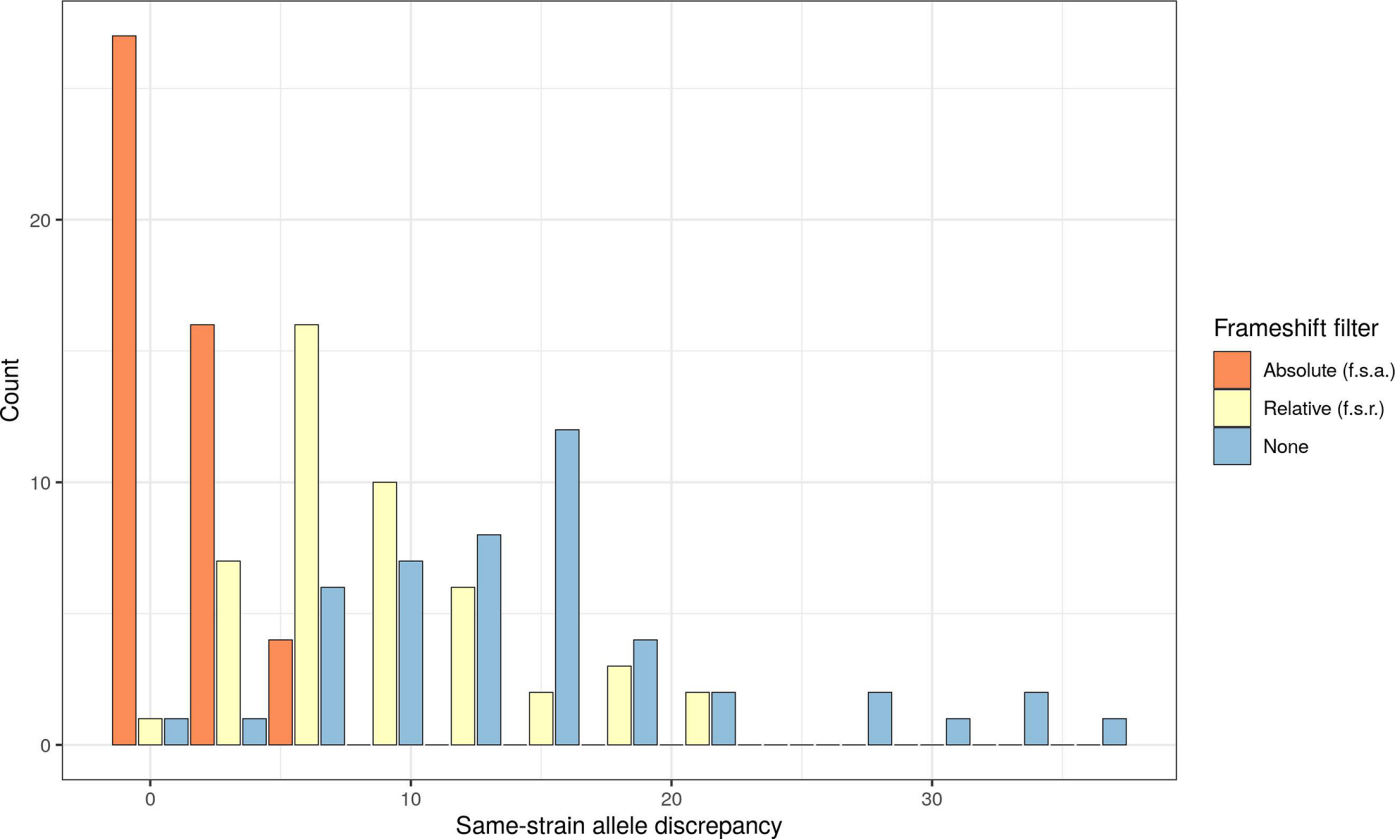
Histogram of the same-strain allele discrepancy in cgMLST analysis after application of no, a relative or an absolute frameshift filter. Increasing the strictness of filtering led to a decrease in the same-strain allele discrepancy.

In cgMLST analysis, the allele distance between the two sequences per isolate was between 1 and 35 (mean 14.5), with no frameshift filter. The frameshift filtering led to a substantial reduction of the same-strain allele discrepancy (Fig. 3). It was more pronounced for the f.s.a. than for the f.s.r. (Figs S1 and S2, available in the online Supplementary Material).

The f.s.a. value decreased the mean same-strain allele discrepancy to 1.7, and the f.s.r. value decreased to 8.5. With no frameshift filter, the fraction of the same sample pairs with an allele discrepancy bigger than 3 was 97.9%. After the application of f.s.r., it decreased to 87.2%, and after the application of the f.s.a., it decreased to 12.8%.

#### cgMLST loci found

**Fig. 4.**
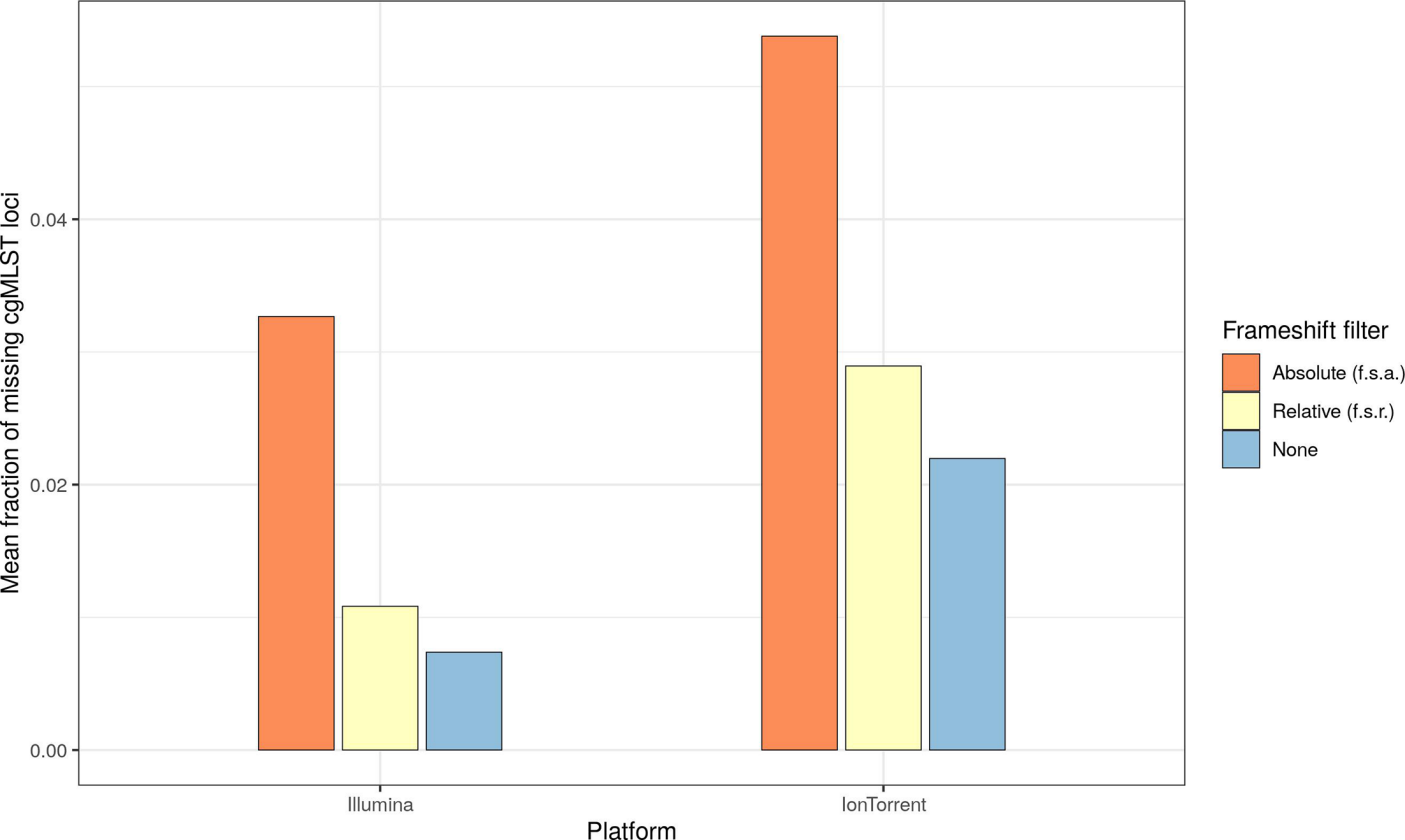
Mean fraction of missing cgMLST loci after the application of no, a relative or an absolute frameshift filter.

Frameshift filtering increased the number of missing cgMLST loci (Fig. 4).

In Illumina data, the mean fraction of missing loci increased from 0.7% (no filter) over 1% (f.s.r.) to 3.3% (f.s.a.), and in Ion Torrent data, it increased from 2.2% (no filter) over 2.9% (f.s.r.) to 5.4% (f.s.a.). The f.s.a. led to the majority of Ion Torrent data (59.6%) missing more than 5% of their loci. However, no samples exhibited more than 10% missing loci. The maximum of missing loci of Ion Torrent data with f.s.a. was 8% (92% of loci found).

#### Clustering benchmark with the seven-allele threshold

Of the 47 isolates, 23 fell into cgMLST clusters with a threshold of ≤7 alleles in single-linkage clustering. Illumina data were defined as the clustering truth. With no frameshift filter, there were no true positives from Ion Torrent data but also no false positives (Fig. S3). Sensitivity was 0%, and specificity was 100%. After the f.s.r., there were four true positives (19 false negatives) and still no false positives (Fig. S4). Sensitivity was 17.4%, and specificity was 100%. After the f.s.a., there were 22 true positives (one false negative) but also one false positive (Fig. S5). Sensitivity was 95.6%, and specificity was 99.9%. In summary, increasing stringency in filtering led to the identification of true positives, while introducing also false positives.

#### Test with known outbreak-related isolates

Nine isolates of the dataset were known to be linked to six listeriosis outbreaks [5,7]. A non-clinical (e.g. food) isolate is considered a match when its distance to a clinical isolate of a listeriosis cluster is ≤7 alleles in cgMLST. The hypotheses formed were confirmed epidemiologically in the case of the nine isolates.

The distance of the non-clinical isolates to the reference sequence of a clinical isolate from a listeriosis cluster was lower for Illumina than for Ion Torrent data (Table 1).

With no frameshift filter, only one of the nine isolates was identified as a match based on the Ion Torrent data. After applying the f.s.r., four of the nine isolates were found to be matches, and after applying the f.s.a., all nine were found to be matches. The distances of Illumina data to the reference sequences of the clinical isolates were stable and independent of the frameshift filters.

#### Cause analysis for discrepancies between Illumina and Ion Torrent

The majority of variants when comparing alleles from Illumina and Ion Torrent data (Ion Torrent sequences with respect to Illumina reference) were deletions (77%), followed by mismatches (13%) and insertions (11.7%). Moreover, missing parts at the beginning or the end of the alignment played a role. Rarely, insertions and deletions occurred in the same alignment (1.8%). The mean length of an insertion was 8 bp (a maximum of 63 bp), and the mean length of a deletion was 38 bp (a maximum of 333 bp). The quantity and length of deletions were notably larger than those of insertions.

Deletions and missing start/end points led to big allele length differences, with mostly longer alleles in the Illumina data. Deletions led to an allele length difference of 3–384 bp (mean: 102 bp) and missing start/end points led to a length difference of 9–408 bp (mean: 167 bp).

**Fig. 5.**
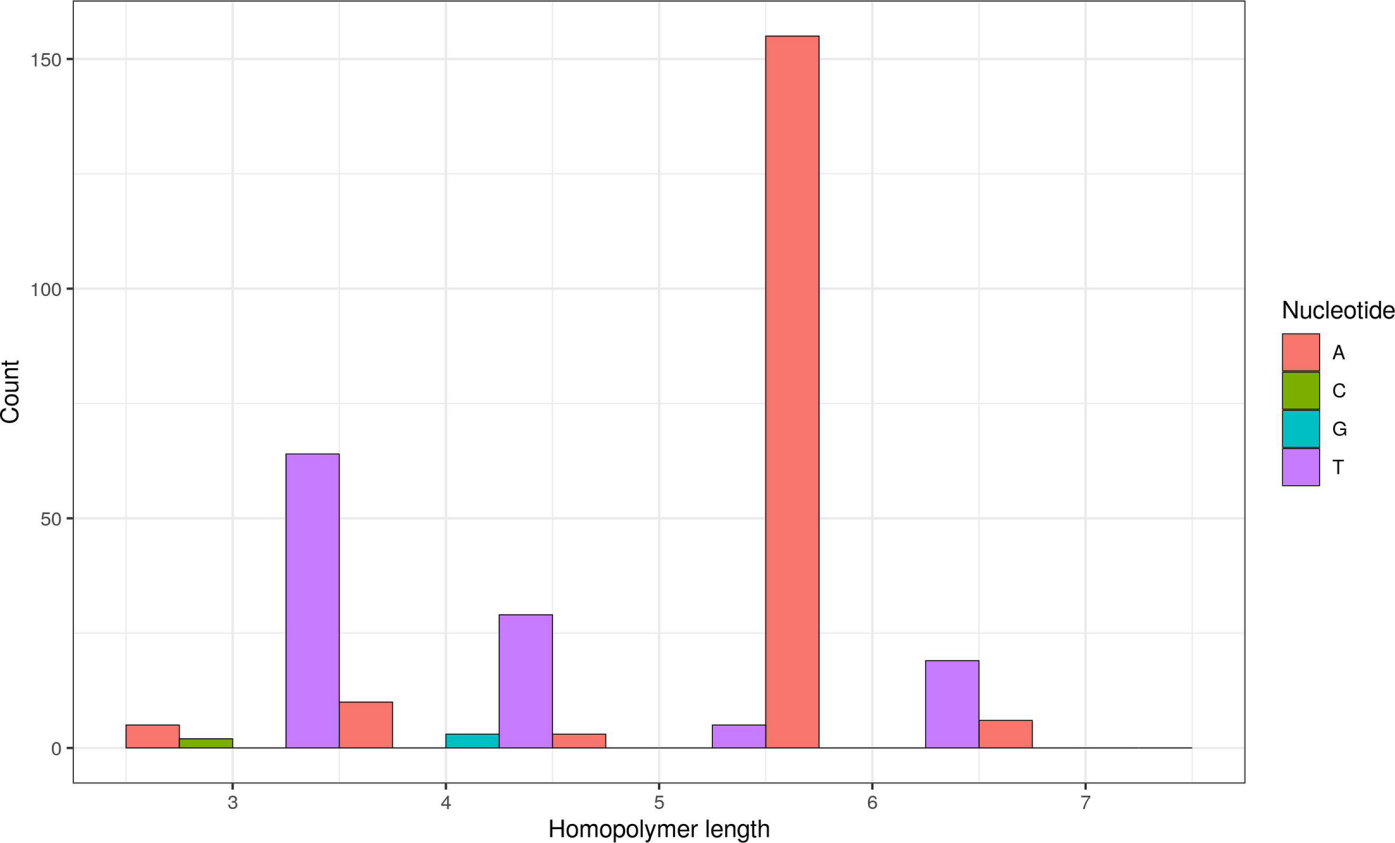
Histogram of homopolymer length for simple InDels comprising a single base only (minimum three consecutive identical bases), per nucleotide, comparing alleles from Illumina and Ion Torrent data (Ion Torrent sequences with respect to Illumina reference).

We checked the distribution of homopolymer length for simple InDels comprising a single base only (minimum of three consecutive identical bases). The mean homopolymer length was 5.5, the maximum value was 9 bp and the most affected nucleotide appeared to be adenine (Fig. 5).

### SNP analysis

There were only minor differences comparing SNP analyses based on raw or trimmed read data (data not shown). As we routinely use trimmed data for SNP analysis, we focused on this approach.

#### Same-strain discrepancy

In SNP analysis, the distance between the two sequences per isolate was between 0 and 47 SNPs (mean: 8; median: 2; Fig. 6).

**Fig. 6.**
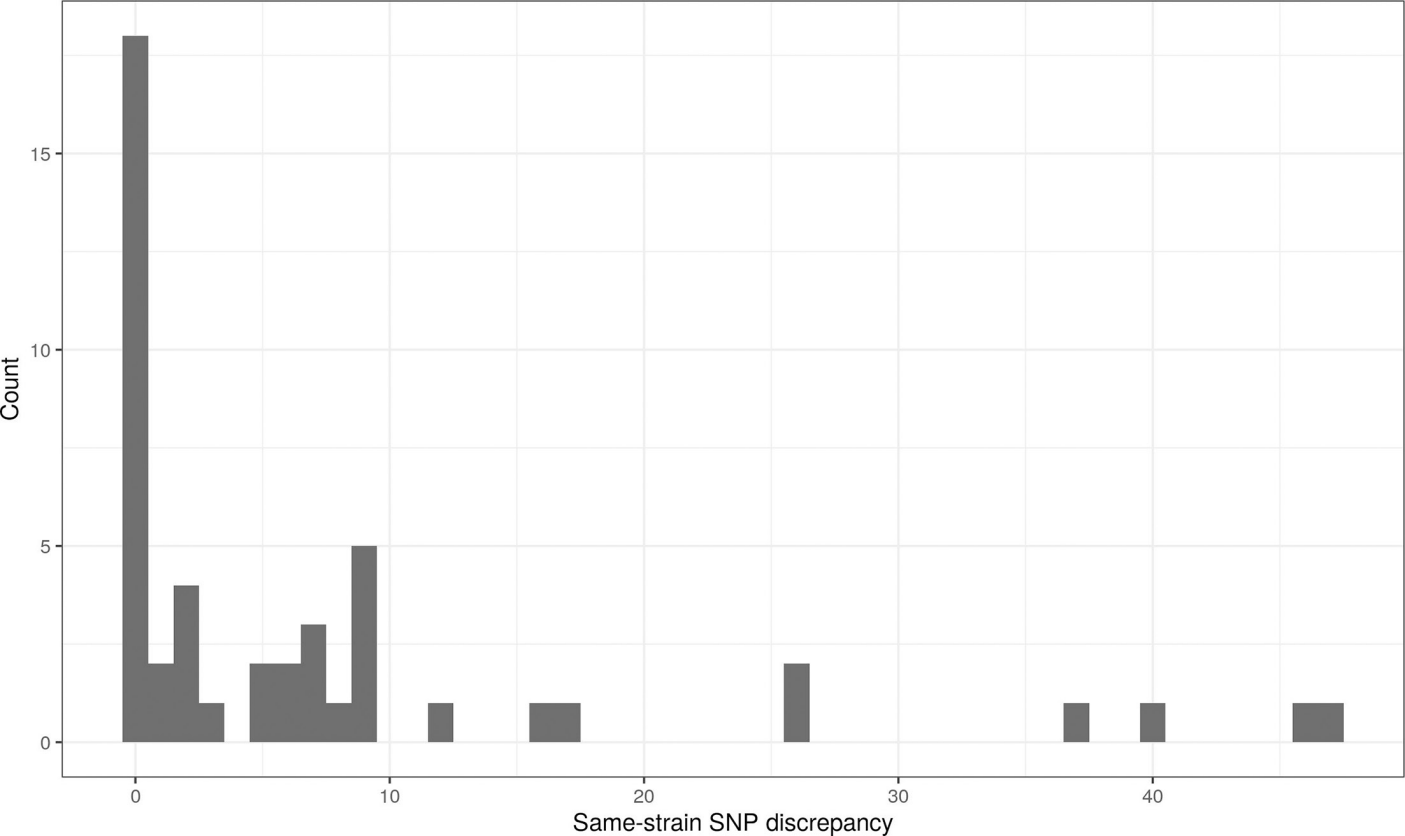
Histogram of the same-strain SNP discrepancy between sequencing data from Illumina and Ion Torrent.

There was no same-strain SNP discrepancy for 18 of the 47 samples (38.3%). Six samples had a discrepancy of more than 20 SNPs.

#### SNPs in Illumina and Ion Torrent data

If SNPs were identified by both platforms, they were highly consistent: 96% of SNP positions (1,225,606 of 1,274,330) were found in both platforms, and all SNPs were identical. Of the remaining positions, only 3% were found in Illumina and only 1% in Ion Torrent data. Accordingly, the types of mutations were very similar between Illumina and Ion Torrent (Fig. S6). The difference is in the heterozygous sites (HETs) before the final variant table (see the following subsection).

#### Core genome size and masking in SNP analysis

An SNP analysis of Illumina data alone showed the biggest core genome size and the lowest number of masked positions (Fig. S7). The core genome size was smaller, and the number of masked positions was larger for Ion Torrent data alone and for mixed Illumina and Ion Torrent data. The number of missing positions (which could not be mapped) appeared to be largely independent of the dataset (Table 2).

**Table 2.** Core genome statistics for SNP analysis. Three different datasets were analysed: Illumina data only, Ion Torrent data only and Illumina and Ion Torrent data combined (=mixed). The reference genome length in all cases was 2,944,528 bp

Analysis	No. of masked positions	No. of missing positions	No. of missing masked positions	Core genome size	Relative core genome size
Illumina only	274,728	420,075	555,737	2,388,791	0.81
Ion Torrent only	1,176,270	401,952	1,391,456	1,553,072	0.53
Mixed	1,267,675	429,032	1,447,651	1,496,877	0.51

Each Ion Torrent sample decreased the core genome size and potentially masked SNPs. The core genome reduction of the entire analysis was bigger than the core genome reduction per sample – meaning that the effect was summed up. The core genome decreased because bases were masked due to HETs. Importantly, the more Ion Torrent data were added to an analysis, the more positions were masked (Fig. S8). As the HETs causing the masking differ from sample to sample, the total number of masked positions increased with the number of samples. This finally led to a reduction of the core genome size available in the SNP analysis. While this holds true for both Illumina and Ion Torrent data, the effect is much more pronounced in the latter. The direct result of the reduced core genome is a reduced resolution of SNP analysis. Reduced core genome means that real SNPs are lost, and SNP distances are systematically underestimated (Figs S9 and S10). The presence of Ion Torrent data in our dataset reduced the SNP distances usually by ~30% (Fig. S9). The relative distance discrepancy was calculated by dividing the difference between the distance of Illumina only and the distance of all data by the distance of Illumina only. For closely related samples (less than 30 SNPs in Illumina-only SNP analysis), this mostly translated into a masking of one to three SNPs. In individual cases, the SNP distance was also reduced from 30 or 28 to 3 SNPs (see Fig. S10). Hence, the presence of Ion Torrent data can pollute an SNP analysis, and this can lead to a false positive association of samples to clusters.

## Discussion

The aim of genome comparison analysis is to get information on distances between sequences of bacterial isolates. This information can be taken from cgMLST or SNP analysis. The usual format is a distance matrix that contains either allele or nucleotide distances. Clustering is performed by applying a threshold value. Bacterial isolates in one cluster are genetically closely related, which indicates that there might also be an epidemiological connection. It should be noted that no conclusions can be drawn based on genetic relationships alone and that epidemiological studies are always necessary to confirm or refute WGS-based hypotheses [34].

In the case of national or international outbreaks or food distribution routes, it is often necessary to combine sequence data generated in different laboratories and under different conditions in one analysis to identify possible connections [35]. In particular, different sequencing technologies can pose a challenge [21,25]. As of 2020, the vast majority (82%) of bacterial genome data in the RefSeq database were generated on Illumina devices [14]. Ion Torrent data were rare but overrepresented in the genomes excluded from the database for ‘many frameshifted proteins’. The number of pseudogenes was also often increased. The main reason for this is that Ion Torrent sequencing is prone to indel errors, especially in regions containing homopolymers [18]. The longer a homopolymer region is, the less likely it is that its length will be correctly recognized [19]. This is because the pH difference that the instrument has to detect is not directly proportional to the number of repeated nucleotides. In our study, adenine seemed to be the most commonly affected nucleotide. Homopolymer errors can lead to frameshifts in assemblies, which is particularly problematic in assembly-based downstream analyses.

A high-quality assembly is essential as a starting point for accurate cgMLST analysis [36]. In our study, the more complete the assembly (as few contigs as possible and as high an N50 value as possible), the more cgMLST loci could be found. Poor assemblies can hinder the detection of cgMLST loci and allele calls. Missing loci and allele misclassification can distort the allele differences, leading to incorrect cluster assignments. In our study, we found that for Illumina data, the choice of assembler did not significantly influence the assembly quality or the number of cgMLST loci detected. Even the assembler MEGAHIT, which was primarily designed for use on metagenomic data [27], delivered good results. For Ion Torrent data, only SPAdes was suitable for producing high-quality assemblies suitable for cgMLST analysis.

Ideally, two sequencings of the same bacterial strain should yield identical results. In cgMLST analysis, the same-strain allele discrepancy should be zero. However, in our study, without applying a frameshift filter, the differences between sequencing the same isolate using Illumina and Ion Torrent were so large that the isolate would not be recognized as the same. In fact, the same-strain allele discrepancy was often above the commonly used threshold of seven alleles for clustering, leading to the same isolate being assigned to different clusters when sequenced on different platforms. The clustering of unknown isolates would be correspondingly questionable. Applying an absolute frameshift filter reduced the mean value of the discrepancies below the clustering threshold. While increasing the stringency of the filter led to the identification of true positives, it also introduced false positives.

To simulate a lifelike scenario, we examined the extent to which epidemiologically confirmed connections could be found using the genomic data. Based on sequence data from clinical cases from published outbreaks, food matches were searched for in the dataset. For Illumina data, applying frameshift filters had no impact on the ability to find matches. For Ion Torrent data, all matches could only be recognized using the absolute frameshift filter. Missing matches occurred without a filter or with a permissive one. However, as the strictness of the filter increased, another problem became more important, which was the increasing number of missing loci.

The analysis of the effect of allele length filtering on allele discrepancy reduction and missing loci highlights the trade-off between increased accuracy and decreased resolution. While filtering is desired to improve accuracy, it can lead to an underestimation of true distances and missing clusters. Additionally, filtering renders distances less comparable between sequencing platforms as different numbers of loci are available for comparison. This issue could be addressed by using a distance metric that accounts for the number of available loci.

We found that the same-strain discrepancy after SNP analysis was smaller than after cgMLST analysis. This is partly because SNP analysis, as a read-based method, is not quite as sensitive to frameshifts as assembly-based methods. In addition, SNP analysis already includes a built-in exclusion or masking of HETs. However, including Ion Torrent data led to the masking of more positions, resulting in a reduction of the core genome size and an underestimation of distances. To avoid this problem, it is advisable to repeat an SNP analysis excluding Ion Torrent data to estimate the extent of the distance reduction caused by including Ion Torrent data.

Overall, we observed that distances tended to be overestimated in cgMLST analysis, leading to false negatives (missed matches). In SNP analysis, however, distances were more likely to be underestimated, potentially resulting in false positives (incorrect matches). The main reason for this is the non-systematic errors that occur in Ion Torrent technology, including deletions, insertions and HETs. The non-systematic nature of these errors makes it challenging to apply precisely tailored filters. Despite technological advances, differences between sequencing platforms remain a challenge for genome comparison analysis. It is important to carefully weigh the pros and cons of different analysis methods and filtering options to achieve reliable results.

## Supplementary material

10.1099/mgen.0.001389 Uncited Supplementary Material 1.

10.1099/mgen.0.001389 Uncited Table S1.

## Abbreviations

cgMLST: core genome multi locus sequence typing
f.s.a.: absolute frameshift filter
f.s.r.: relative frameshift filter
HETs: heterozygous sites
WGS: whole-genome sequencing
