# Supplementary material for: Compatibility of whole-genome sequencing data from Illumina and Ion Torrent technologies in genome comparison analysis of Listeria monocytogenes
Source: Microb Genom. 2025 May 1;11(5):001389. doi: 10.1099/mgen.0.001389 (PMC12046094; doi:10.1099/mgen.0.001389)
Supplement: Uncited Supplementary Material 1. [file mgen-11-01389-s001.pdf]

Manuscript Supplement

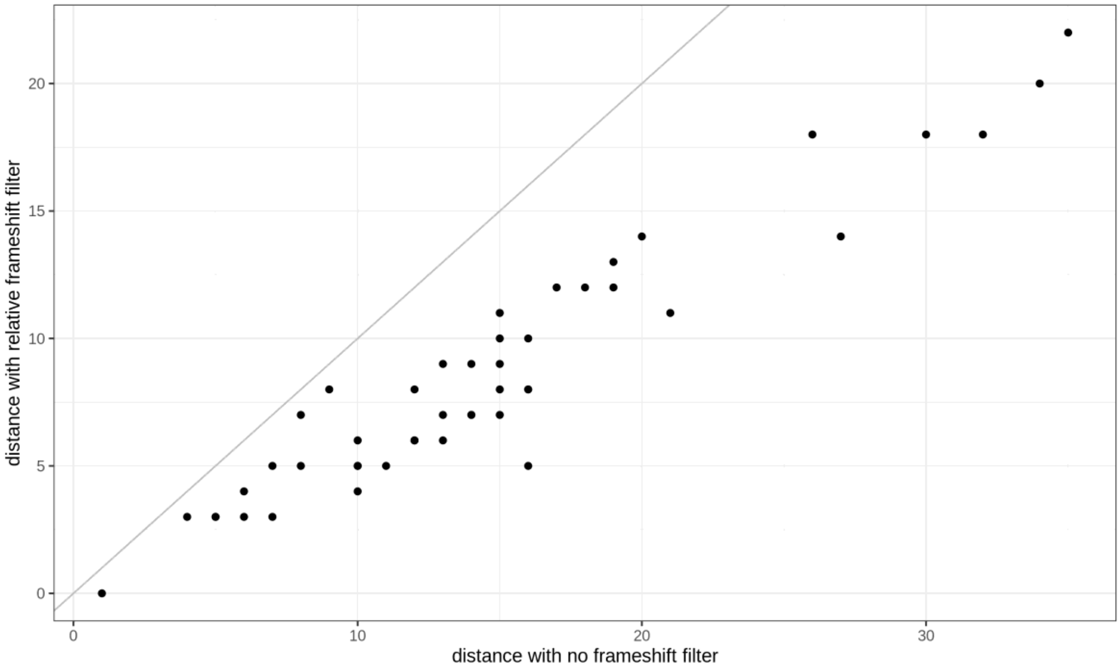

Figure 1: Scatterplot of pairwise allele distances in closely related samples of the dataset with no and with a relative frameshift filter in cgMLST analysis. Application of a relative frameshift filter tends to lower the pairwise allele distance.

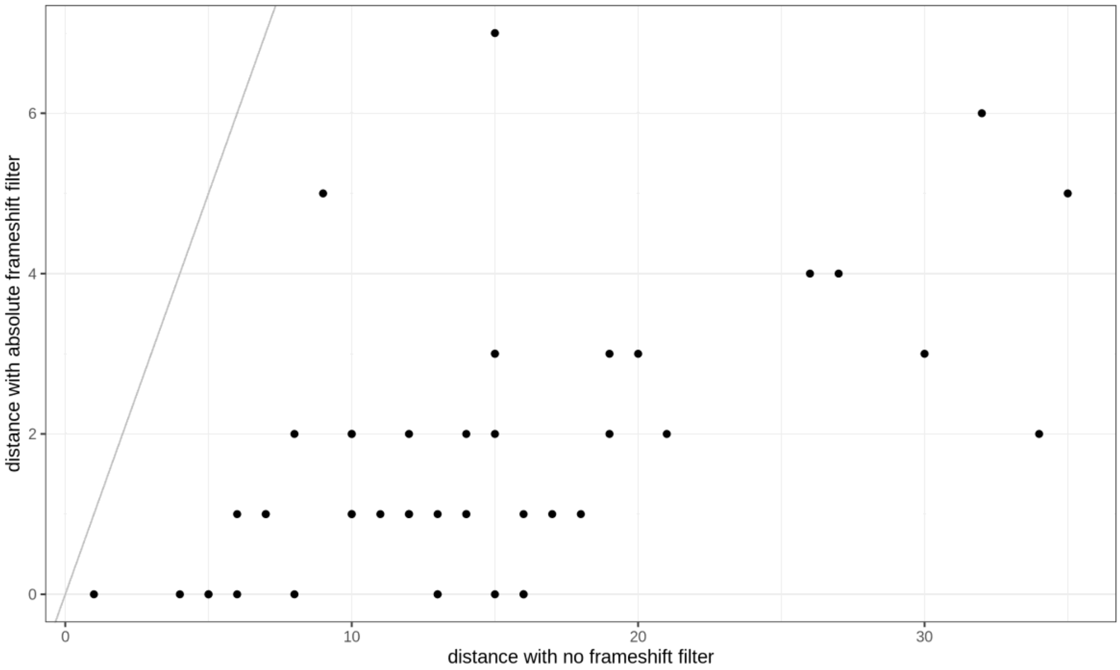

Figure 2: Scatterplot of pairwise allele distances in closely related samples of the dataset with no and with an absolute frameshift filter in cgMLST analysis. Application of an absolute frameshift filter tends to lower the pairwise allele distance.

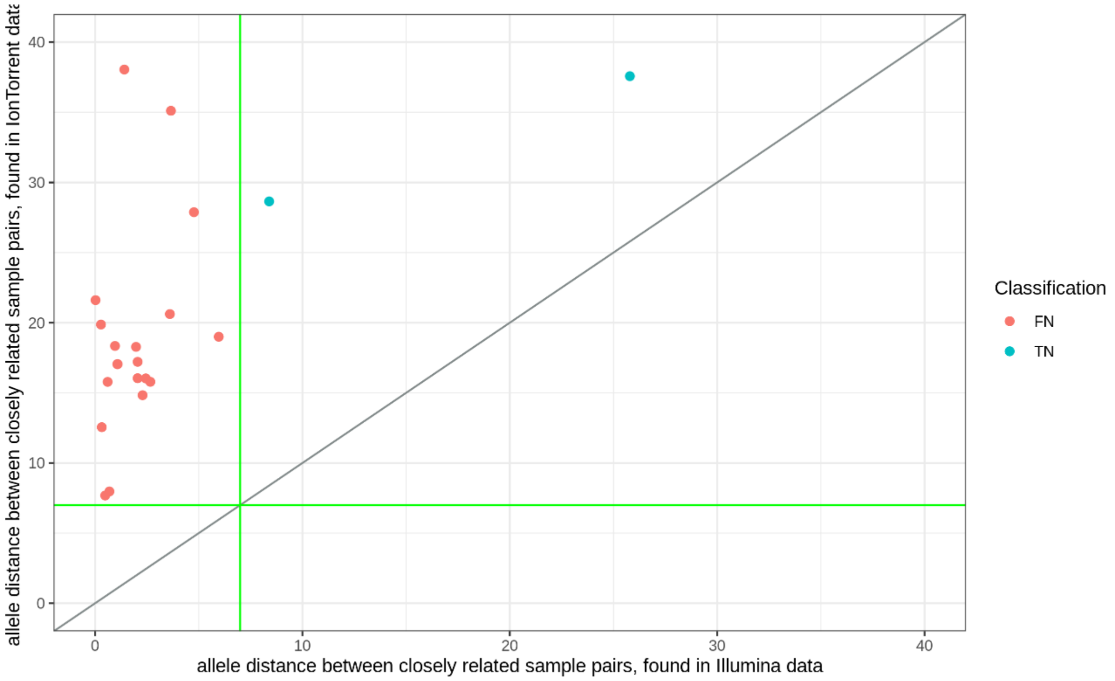

Figure 3: Sensitivity and specificity in cgMLST analysis of Ion Torrent and Illumina data, with no frameshift filter. FN: false negative, TN: true negative. The horizontal and vertical green lines indicate the threshold value of seven allele differences, which is commonly used for clustering.

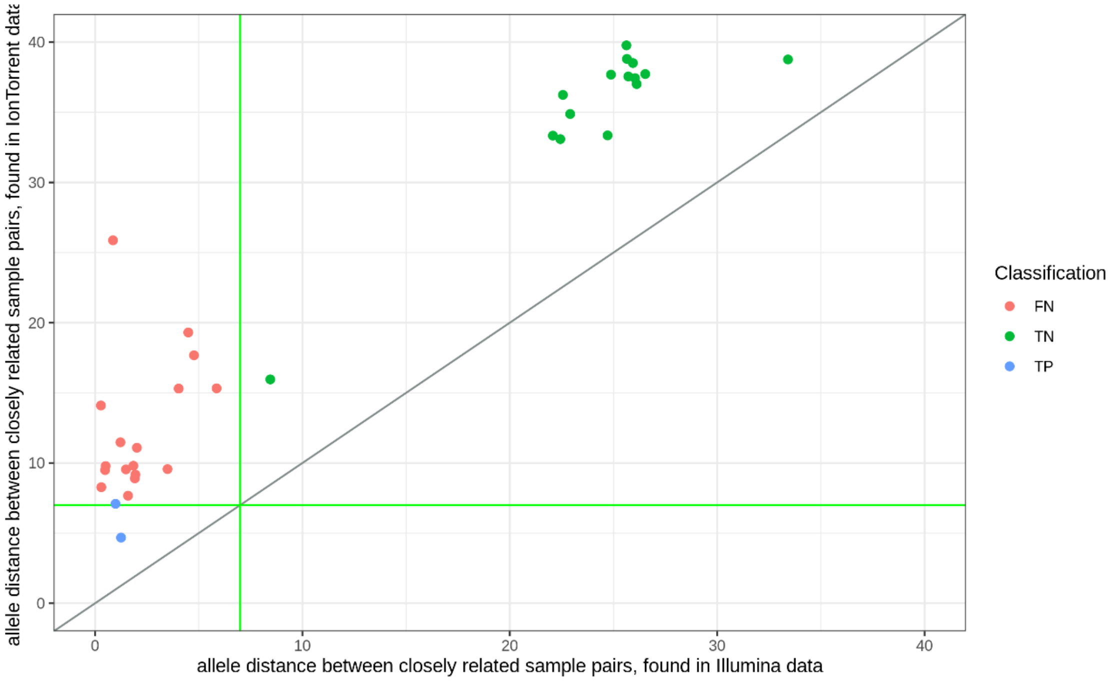

Figure 4: Sensitivity and specificity in cgMLST analysis of Ion Torrent and Illumina data, with a relative frameshift filter. FN: false negative, TN: true negative, TP: true positive. The horizontal and vertical green lines indicate the threshold value of seven allele differences, which is commonly used for clustering.

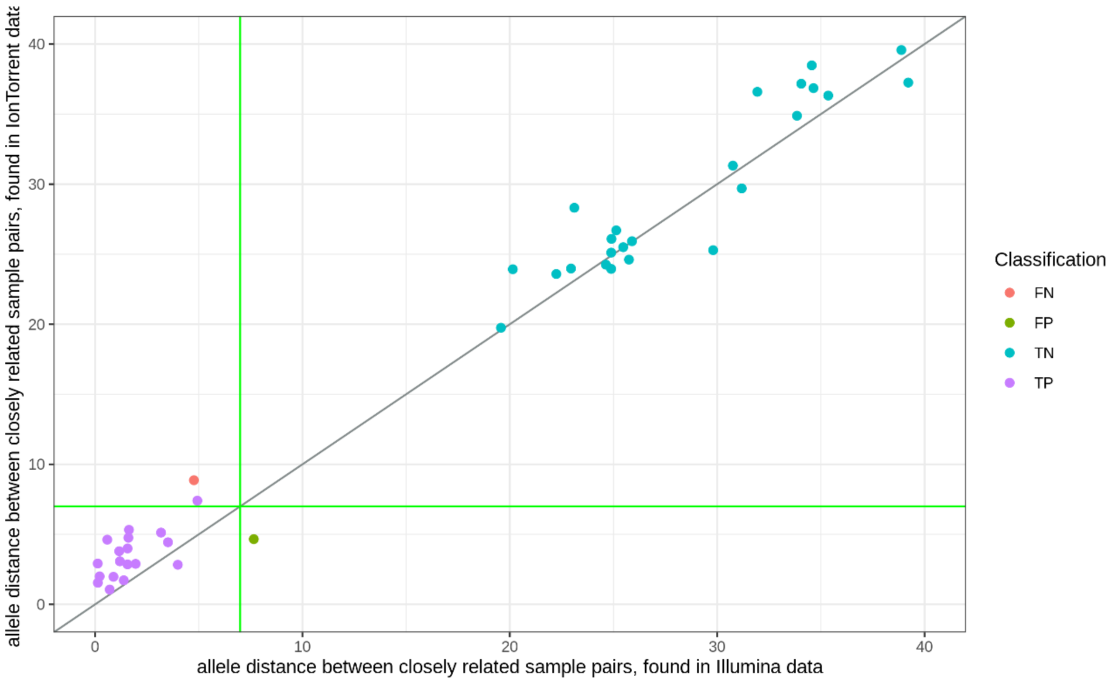

Figure 5: Sensitivity and specificity in cgMLST analysis of Ion Torrent and Illumina data, with an absolute frameshift filter. FN: false negative, FP: false positive, TN: true negative, TP: true positive. The horizontal and vertical green lines indicate the threshold value of seven allele differences, which is commonly used for clustering.

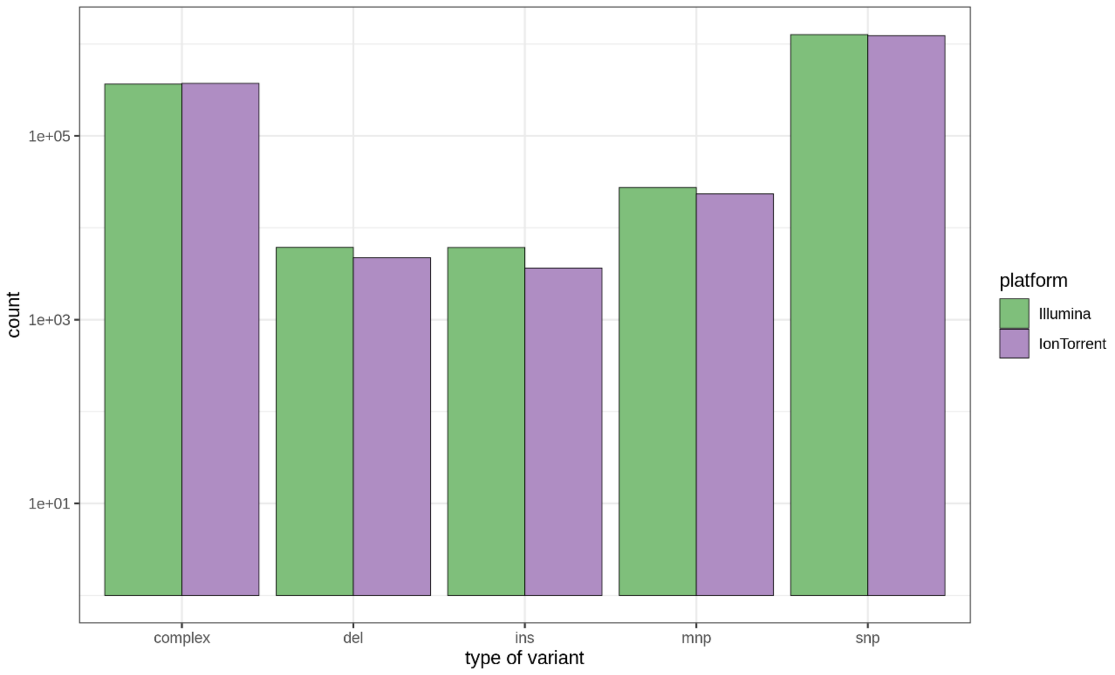

Figure 6: Number of variant types found in SNP analysis of data from Illumina and Ion Torrent. Del: Deletion, Ins: Insertion, Mnp: Multi nucleotide polymorphism, SNP: single nucleotide polymorphism.

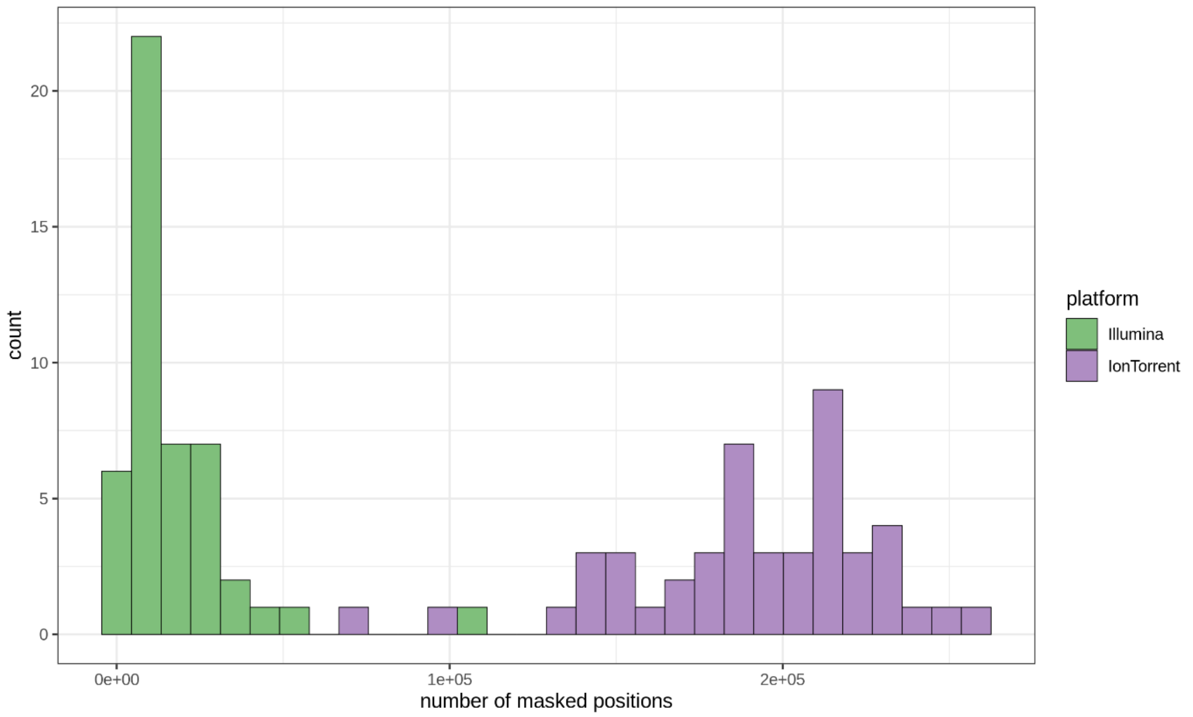

Figure 7: Histogram of the number of masked positions in SNP analysis of Illumina and of IonTorrent data. In IonTorrent data, on average, more positions are masked.

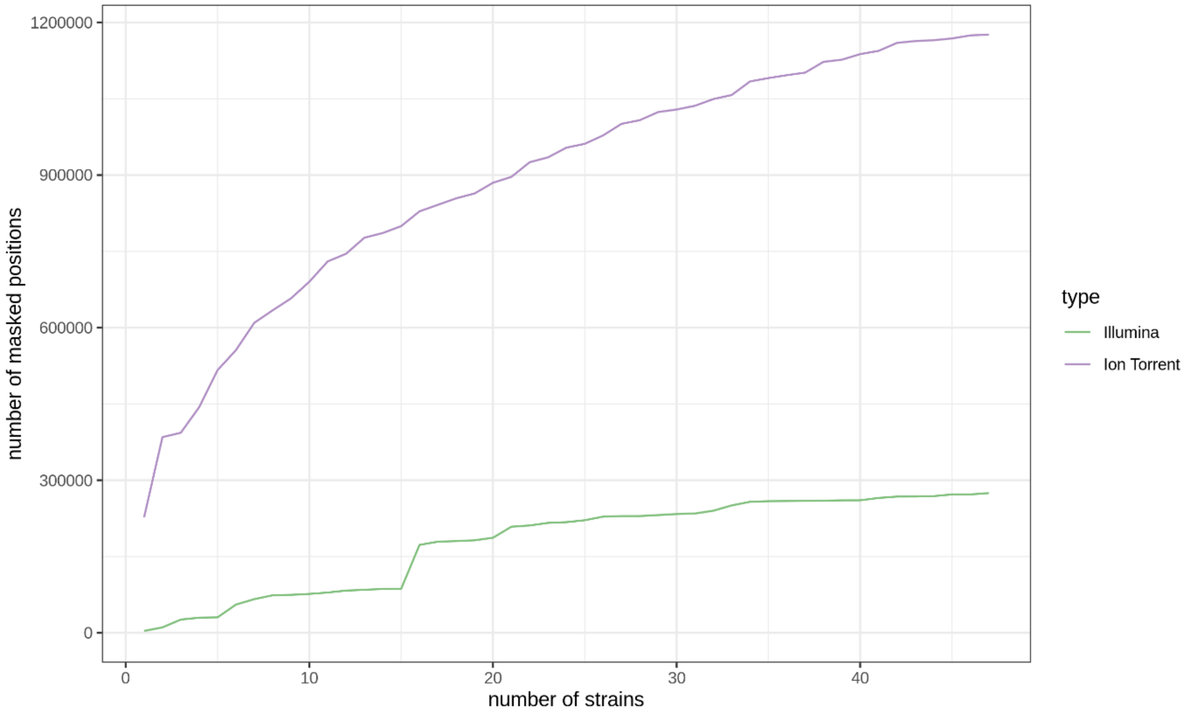

Figure 8: Cumulative masking effect in SNP analysis ((Illumina and Ion Torrent data together in one analysis). The order of the strains was shuffled. Masked positions were counted (by type they came from, Illumina or Ion Torrent) while adding more and more samples. As the masked positions differ from sample to sample, the total number of masked positions increases with the number of samples.

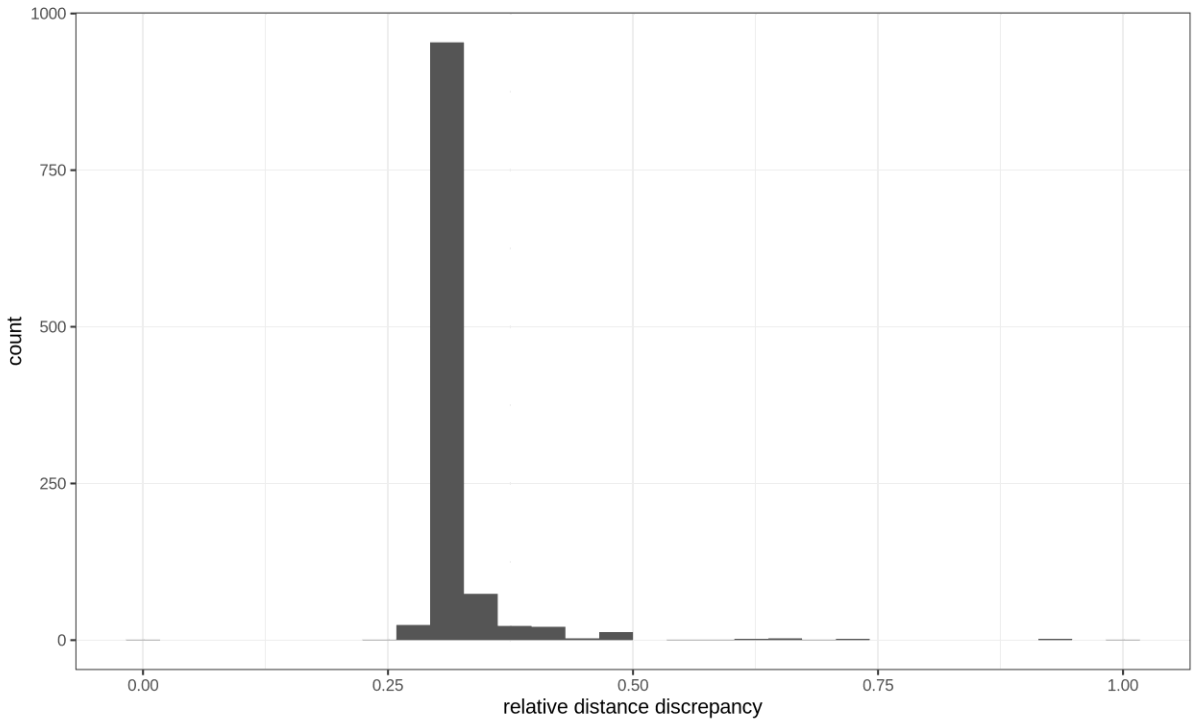

Figure 9: Histogram of relative distance discrepancy for pairwise Illumina distances with and without Ion Torrent data. The relative distance discrepancy is defined as (Distance.illuminaonly - Distance.alldata)/Distance.illuminaonly.

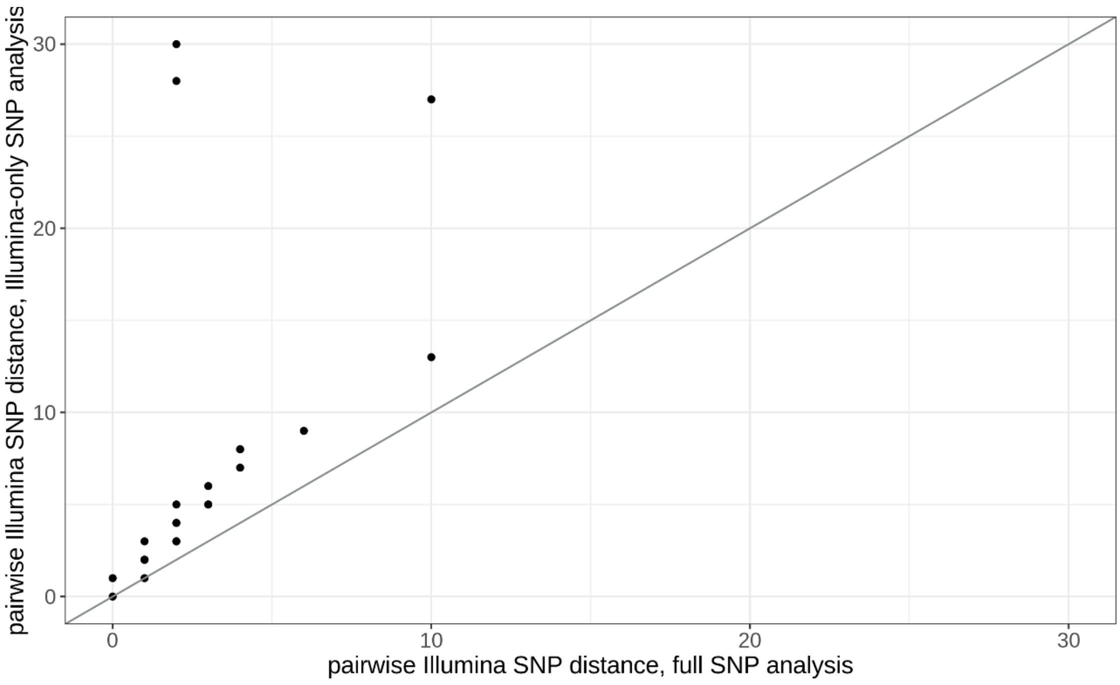

Figure 10: Scatterplot of pairwise SNP distances in closely related samples with (full SNP analysis) and without (Illumina-only SNP analysis) inclusion of IonTorrent data in SNP analysis. Inclusion of IonTorrent data in the analysis tends to lower the pairwise SNP distances.
